# Development and evaluation of a Hospice Foundation of Taiwan Bereavement Assessment Scale: A psychometric properties test

**DOI:** 10.1017/S1478951524001706

**Published:** 2025-01-27

**Authors:** Te-Yu Wu, Shih-Hsuan Pi, Pei-Yi Li, Yuen-Liang Lai, Chin-Ching Li, Chun-Kai Fang

**Affiliations:** 1 Department of Nursing, MacKay Medical College, New Taipei, Taiwan; 2 Department of Nursing, MacKay Memorial Hospital, Taipei, Taiwan; 3 Department of Medical Research, Tamsui Branch, MacKay Memorial Hospital, New Taipei, Taiwan; 4 General Education Center, MacKay Junior College of Medicine, Nursing, and Management, New Taipei, Taiwan; 5 Department of Crime Prevention, Central Police University, Taoyuan, Taiwan; 6 Department of Thanatology and Health Counseling, National Taipei University of Nursing and Health Sciences, Taipei, Taiwan; 7 Center of Holistic Education, MacKay Medical College, New Taipei, Taiwan; 8 Hospice and Palliative Care Center, Tamsui Branch, MacKay Memorial Hospital, New Taipei, Taiwan; 9 Hospice and Palliative Care Center and Department of Psychiatry, MacKay Memorial Hospital, Taipei, Taiwan; 10 Department of Medicine, MacKay Medical College, New Taipei, Taiwan; 11 Department of Death Care Service, MacKay Junior College of Medicine, Nursing and Management, Taipei, Taiwan

**Keywords:** Grief, bereavement, palliative care, psychometric properties, family caregivers

## Abstract

**Objectives:**

Supporting family caregivers (FCs) is a critical core function of palliative care. Brief, reliable tools suitable for busy clinical work in Taiwan are needed to assess bereavement risk factors accurately. The aim is to develop and evaluate a brief bereavement scale completed by FCs and applicable to medical staff.

**Methods:**

This study adopted convenience sampling. Participants were approached through an intentional sampling of patients’ FCs at 1 palliative care center in Taiwan. This cross-sectional study referred to 4 theories to generate the initial version of the Hospice Foundation of Taiwan Bereavement Assessment Scale (HFT-BAS). A 9-item questionnaire was initially developed by 12 palliative care experts through Delphi and verified by content validity. A combination of exploratory factor analysis (EFA), reliability measures including items analysis, Cronbach’s alpha and inter-subscale correlations, and confirmatory factor analysis (CFA) was employed to test its psychometric properties.

**Results:**

Two hundred seventy-eight participants conducted the questionnaire. Three dimensions were subsequently extracted by EFA: “Intimate relationship,” “Existential meaning,” and “Disorganization.” The Cronbach’s alpha of the HFT-BAS scale was 0.70, while the 3 dimensions were all significantly correlated with total scores. CFA was the measurement model: chi-squared/degrees of freedom ratio = 1.9, Goodness of Fit Index = 0.93, Comparative Fit Index = 0.92, root mean square error of approximation = 0.08. CFA confirmed the scale’s construct validity with a good model fit.

**Significance of results:**

This study developed an HFT-BAS and assessed its psychometric properties. The scale can evaluate the bereavement risk factors of FCs in clinical palliative care.

## Introduction

Bereavement is a period of mourning after a loss, which can be disruptive for some (Sanderson et al. 2022). Disturbed grief, such as prolonged grief disorder or persistent complex bereavement disorder, is associated with an increased risk of several psychiatric disorders (Boelen and Smid 2017), promotes inflammation following acute stress (Brown et al. 2022), and most will experience it during their lifetime (Sanderson et al. 2022). This occurs in 10% of bereaved persons, including spouses, children, and in those who continue to suffer after the first 6–12 months of bereavement (Boelen and Smid 2017).

Family caregivers (FCs) are essential in palliative care. Palliative care is a comprehensive model that aims to improve patients’ and their families’ quality of life and to alleviate life-threatening illnesses via the early detection, assessment, and treatment of health-associated problems (World Health Organization 2002). Therefore, support for FCs, including bereavement follow-up, is a core function of palliative care. However, only 39.4% of the bereaved were debriefed about their emotional and psychological distress pre-bereavement while only half perceived support was sufficiently provided by palliative care services. Many FCs have expressed unmet needs, requiring more information, preparation, and assistance (Hudson et al. 2012). Therefore, it should be worthwhile to invest palliative care efforts in assessing and supporting FCs during the anticipatory grief period (Aoun et al. 2017). Providing the same psychosocial support to all FCs receiving palliative care is not enough. Additionally, the palliative care teams need to find FCs who may have complicated grief in the future and provide more psychosocial support (Hudson et al. 2012). Therefore, FCs should be offered assessments and access to relevant psychosocial support. Improvement in bereavement care practice is of high priority and risk assessment is an essential procedure for the provision of bereavement support (Sealey et al. 2015).

The following bereavement/grief assessment tools are currently employed In palliative care: Bereavement Risk Assessment Tool (BRAT) (Rose et al. 2011), Bereavement Risk Index (Kristjanson et al. 2005), Marwit-Meuser Caregiver Grief Inventory (MMCGI) (Marwit and Meuser 2005), Anticipatory Grief Scale (Holm et al. 2019), the Caregiver Grief Scale (Meichsner et al. 2016), prolonged grief (Coelho et al. 2017), Grief Resolution Index (Remondet and Hansson 1987), and Texas Revised Inventory of Grief (Futterman et al. 2010). We have listed 8 tools currently available for bereavement/grief assessment. Despite their excellent reliability and validity in assessing FCs’ bereavement/grief, these tools are often more the cultural differences inherent in tools developed within Western cultural considerations. Bereavement is a normal reaction to the death of a loved one. However, that is an experience heavily influenced by traditions and culture. While the majority of individuals adapt to their loss without professional disposal, grieving remains an intense, painful, and isolating period for many, often enduring months or even years (Grassi and Riba 2012). It has long been recognized as an essential role of culture in framing the mourning experience as a process of meaning reconstruction and relationship (Silverman et al. 2021). Research has shown that cultural differences can significantly impact the expression and experience of grief (Boelen and Smid 2017). Therefore, this may lead to discrepancies in the effectiveness of bereavement tools when applied in non-Western settings.

Since bereavement is an experience heavily influenced by culture, as clinicians, professional culture and personal backgrounds play a role in determining how we understand and respond to others’ grief. Moreover, many scales with good reliability and validity have been developed and copyrighted for financial considerations, which can hinder their widespread use in Taiwan. Therefore, the Hospice Foundation of Taiwan Bereavement Assessment Scale (HFT-BAS) was originally an academic seminar held by HFT on World Hospice Day in 2015. On that day, many palliative care clinicians reported that taking into account local culture and medical capacity, they were requested to develop a clinical rapid assessment of bereaved families instrument. Screening evaluations are administered before a patient’s death for FCs of Taiwan. Therefore, this study aims to develop and evaluate the psychometric properties of the HFT-BAS scale to predict bereavement risk factors in palliative care for FCs.

## Methods

This was a cross-sectional study designed according to the 3 stages of scale development: item development (Phase 1), scale development (Phase 2), and scale evaluation (Phase 3) (Boateng et al. 2018).

### Phase 1. Item development

The scale was developed through literature reviews on grief and bereavement. Grief is a syndrome with psychological and bodily symptomatology. It may appear immediately or be delayed after a crisis; it may be exaggerated or apparent. If the bereavement occurs at a time when the patient is faced with essential tasks and when there is a necessity for maintaining the morale of others, he may show little or no reaction for weeks or even much longer (Lindemann 1944). However, bereavement is the period after a loss during which grief occurs. The time spent in bereavement for the loss of a loved one depends on the situation of the loss and the level of attachment to the person who died (Casarett et al. 2001). Since 2004, Taiwan has accumulated a lot of knowledge and clinical experience on Western grief theories, thanks to the efforts of 2 organizations, the Taiwan Hospice Foundation and the Department of Death and Health Counseling at the National Taipei College of Nursing and Health (Fan et al. 2019; Lu et al. 2007). After more than 20 years of experience in contact with Western grief theories, clinicians in Taiwan’s palliative care particularly agree with Family Focused Grief Therapy (FFGT), Two Track Model (TTM), Dual Process Model (DPM), and Meaning Reconstruction Model (MRM). We integrated 4 theories from Western and Eastern cultures and philosophies to design the HFT-BAS. FCs’ grief and bereavement were measured, producing a simple scale suitable for clinical application.

1. FFGT: Because of caring the feelings the whole family, FFGT’s family view agrees with Chinese values and has been employed while the patient was alive, which conforms with Taiwan’s clinical service model. FFGT has a matching tool, the Family Relationships Index, with 12 questions, including 3 subscales – “cohesiveness,” “expressiveness,” and “reversed conflict” (Kissane and Bloch 2002). The first 3 questions of HFT-BAS were based on the above 3 subscales.
2. TTM: The central concept of the theory is that the bereaved family will continue to live on 2 tracks, that is, maintaining biopsychosocial functioning and a solid connection to the deceased (Rubin 2010). Questions 4 and 5 of HFT-BAS were designed from these 2 aspects.
3. DPM: DPM describes the experience of many grieving people well. The DPM suggests that bereaved persons oscillate between loss-oriented and restoration-oriented stressors (Stroebe and Schut 1999). According to these 2 aspects of DPM, questions 6 and 7 of HFT-BAS were formulated.
4. MRM: The theory comprises 2 aspects from which questions 8 and 9 of HFT-BAS were derived – losing the meaning of life upon a loved one’s death and recreating life’s meaning for the future (Neimeyer 2000).

Overall, the HFT-BAS consisted of 3 questions from the FFGT, 2 from the TTM, and a 9-item scale compiled from DPM and MRM theories, asking whether the subjects had cared for family members in the last 6 months. Further, a 4-point Likert scale was adopted, with 1–4 points representing “Never,” “Sometimes,” “Often,” and “Always.” Total scores range from 9–36, among which items 1, 2, 5, 7, and 9 were reverse scores, whereas items 3, 4, 6, and 8 were positive. The higher the score, the higher the degree of bereavement.

After the initial scale was developed, 6 palliative care experts, which included 3 professors in the field of palliative care, a head nurse, a social worker, and a professor of grief counseling, were invited to evaluate its content validity. The initial questionnaire was applied anonymously using the Delphi method. An additional 6 experts, 4 palliative care physicians, and 2 professors were invited to provide their opinions on each item, after which they exchanged views with the original 6 palliative care experts. This cycle was repeated 3 times until the questionnaire was finalized. All 6 experts agreed with the concepts of all 9 questions for the first time, but they still had slightly different opinions on the wording. By the third time, the proportion of each suitable question had reached 100%. Content validity was assessed by evaluating the “relevance” of each item through a Likert scale, ranging from 1 = not relevant, 2 = somewhat relevant, 3 = quite relevant, to 4 = highly relevant. This strategy was employed to calculate the item-content validity index (I-CVI), where a minimum of .78 was acceptable (Lynn 1986) and a minimum scale-content validity index (S-CVI) of .80 was recommended (Davis 1992). The 9-item average I-CVI on this scale was 0.86, while the S-CVI was 0.83. In addition, all items must achieve a relevance rating of 3 or 4 by all the experts. The 4-point item was then partially revised after consultation with experts.

### Phase 2: Scale development

After 2 meetings to modify the sentences, the experts agreed to retain all the items and to conduct a pilot test. Five middle school and university graduates were recruited to administer the test to terminal cancer FCs. To confirm participants’ readability and comprehension of each item, a 4-point scale was developed where 1 = completely disagree, 2 = disagree, 3 = agree, and 4 = completely agree (Terwee et al. 2007), followed by psychometric properties tests of the initial version scale.

A cross-sectional study was designed to collect data on palliative care FCs in medical center hospitals and subsequently test the reliability and validity of the scale. Reliability was evaluated via item analysis, Cronbach alpha, and item-total score correlation, while validity used exploratory factor analysis (EFA) and confirmatory factor analysis (CFA). Item analysis, skewness/standard error (SE), kurtosis/SE kurtosis, and extreme group comparisons were assessed via mean and standard deviation (SD) of continuous, normally distributed variables to determine normality and discrimination. The critical ratio (CR) was utilized to select the 27^th^ and 73^rd^ percentile cut-off points, representing the low and high groups, respectively, followed by independent *t*-tests to determine the degree of discrimination of each item (Kelley 1939). On the other hand, EFA included the Kaiser-Meyer-Olkin test for sampling adequacy and Bartlett’s test of sphericity. The extraction method was mainly based on principal component analysis. The varimax rotation method and the screen plot were used as the standard for extracting factors (Strickland 2003). In addition, construct validity was assessed using CFA with a good model fit to linear structural relation (LISREL), the chi-square (χ^2^) value, root mean square error of approximation (RMSEA), and supplement of comparative fit index (CFI) were calculated to assess an adequate model.

### Phase 3: Scale evaluation of the final version of HFT-BAS

#### Participants

This study adopted convenience sampling, wherein samples were obtained through intentionally sampling patients’ FCs at the palliative care center between May 2016 and October 2017. Inclusion criteria were as follows: (1) adults over 20 years old; (2) effectively communicate in Mandarin Chinese; (3) agree and have signed the consent form; and (4) clear mental state. Exclusion criteria were as follows: (1) not primary caregivers and 2) cognitive impairment. A ratio for minimal sample size: item:cases = 1:5 (Comrey 1988). In this study, the cases 5*9 = 45 should be adequate.

## Ethical considerations

The Research Ethics Committee of the Institutional Review Board approved this study (16MMHIS028). The researchers explained the study purpose and participant rights and obtained written consent from all participants in a private single room. Participants may terminate their participation in the study at any time.

## Data processing and statistical analysis

The questionnaire’s incomplete information was deleted. This study employed SPSS version 22.0 to organize and code the data, reliability analysis, EFA, and LISREL version 8.83 (Scientific Software International, Inc., Skokie, IL) as validation CFA.

## Results

### Demographic characteristics of the participants

Two hundred and seventy-eight participants arrived at the palliative care center in Taiwan between 2016 and 2017, where they completed the questionnaire and data was analyzed. The participant demographics are summarized in Table 1.

**Table 1.** Demographic characteristics of the participants (*n* = 278)

Characteristic	Mean ± SD	*N* (%)
Age	46.9 ± 14.5	
Gender		
Man		108 (38.9)
Female		168 (60.4)
Missing		2 (0.7)
Religious beliefs		
Yes		194 (69.7)
None		63 (22.7)
Missing		21 (7.6)
Education		
High school (below)		143 (51.5)
College degree (above)		133 (47.8)
Missing		2 (0.7)
Employment status		
Employed		181 (65.1)
Unemployed		94 (33.8)
Missing		3 (1.1)

### Tests of reliability: Item analysis and item total correlation

The average HTF-BAS score of this study was 21.1 ± 3.4 (Table 2). The Skewness and kurtosis scores were 0.14 and 0.29, indicating that the comparison of skewness and kurtosis was normal. The CR test represented the low (2.90) and high groups (3.64), and conducted an extreme group mean difference test (independent *t*-test), respectively showing the more discriminative between items (except Q3). Additionally, the Pearson correlation coefficient between the item-total score correlation was between 0.39 and 0.53 and has significance (except Q3), these results indicated that all items contribute to the HFT-BAS (Kelley 1939; Polit and Beck 2012). Therefore, Q3 should be removed since there was no significant distinction between other items. Since the majority of participants in this study were female (60.4%). One study showed that 25.9% of FCs had a high (16.1%) to severe (9.8%) level of pre-loss grief, higher degrees in women than in men (Treml et al. 2021). Therefore, after discussions with experts, this question was retained due to the average score being the highest among the participants, and the measurable family members having a high degree of bereavement (Table 2). Additionally, this study uses a single sample for statistical analysis. First, a sample Kolmogorov–Smirnov test for normal distribution is performed with *p* = 0.004. The result is *p* < 0.05, which rejects the null hypothesis. It shows that there is no normal distribution of sample scores, so the median value is 21 points, and 41% still accounted for 21 points and above, which should be why medical personnel should pay more attention to ethnic groups as a high risk.

**Table 2.** Item analysis of HFT-BAS (*n* = 278)

			Skewness		Kurtosis			95%confidence interval		Item correlation
Questionaire items	Mean	SD	Statistic	Std. error	Statistic	Std.error	*t*	Lower	Upper	with total score
Total score of scale	21.1	3.4								
Q 1.	1.6	0.7	1.004	0.146	0.087	0.291	−9.26***	−1.35	−0.87	0.43**
Q 2.	1.7	0.7	0.684	0.146	−0.663	0.291	−8.84***	−1.29	−0.82	0.44**
Q 3.	3.3	0.7	−0.711	0.146	0.919	0.291	−0.54	−0.34	1.96	0.09
Q 4.	3.2	1.0	−0.904	0.146	−0.367	0.291	−6.11***	−1.54	−0.78	0.39**
Q 5.	2.0	0.9	0.472	0.147	−0.813	0.293	−10.12***	−1.77	−1.19	0.53**
Q 6.	2.9	0.9	−0.651	0.146	−0.597	0.291	−4.85***	−1.29	−0.54	0.53**
Q 7.	1.9	1.0	0.834	0.146	−0.534	0.291	−10.76***	−1.96	−1.35	0.51**
Q 8.	3.2	0.9	−0.959	0.146	−0.018	0.291	−4.84***	−1.15	−0.48	0.51**
Q 9.	1.3	0.6	2.107	0.146	4.866	0.291	−6.63***	−1.03	−0.55	0.47**

** At a significance level of 0.05 (2-tailed), the correlation was significant.

*** At a significance level of 0.01 (2-tailed), the correlation was significant.

### EFA and Cronbach’s alpha

Additionally, the sample (278 cases) uses a random number generator to split into 2 independent samples (every 139 cases were to groups A and B) to conduct EFA and CFA individually. Before EFA, the reverse questions 1, 2, 5, 7, and 9 were converted to forward scoring. The Kaiser-Meyer-Olkin score for the 9 questions was 0.645, while Bartlett’s test of sphericity showed a significant difference (*p* < 0.000); thus, further EFA could be conducted. Three factors were extracted with a factor loading of 0.52–0.89 (Table 3), and the cumulative explained variance of these 3 factors reached 60.15%.

**Table 3.** Rotated factors for principal components analysis of HFT-BAS (*n* = 139 (group A))

	Factor loading		
Questionnaire items	I	II	III
**Factor 1: Intimate relationship**			
1. Family members can easily get help from each other.	0.731		
2. Family members are often able to communicate with each other well.	0.697		
3. Frequent conflicts between family members.	0.666		
**Factor 2: Existential meaning**			
5. I can talk to patients about everything and enjoy every moment.		0.613	
7. Even in the face of disease uncertainties for patients, I will know how to live.		0.753	
9. What I’m doing for patients now feels meaningful to me.		0.686	
**Factor 3: Disorganization**			
4. I lost time with other family members or my job due to caring for patients.			0.621
6. I’m always worried that I won’t know how to deal with my loved ones if they pass away.			0.875
8. If a loved one dies, I will lose my life’s purpose and meaning.			0.866

This study extracted 3 subscales through EFA, of which “**Disorganization**” had the highest average score. Cronbach’s alpha of the HFT-BAS was 0.70, while Cronbach’s alpha of the total score of the 3 subscales was 0.50–0.82. In addition, Spearman’s correlation coefficient between the 3 factors and the total score ranged between 0.50 and 0.70, with a significant correlation to the total score (Table 4).

**Table 4.** Reliability of the HFT-BAS (*n* = 139 (group A))

Components	Mean ± SD	Cronbach’s α	Correlation between 3 factors and total score
Total score of the HFT-BAS scale	21.6 ± 3.3	0.70	
Factor 1: Intimate relationship	6.9 ± 1.2	0.50	0.56***
Factor 2: Existential meaning	5.5 ± 1.9	0.82	0.70***
Factor 3: Disorganization	9.5 ± 2.0	0.65	0.50***

*** *p* < 0.001.

### Tests of construct validity: CFA

**Figure 1.**
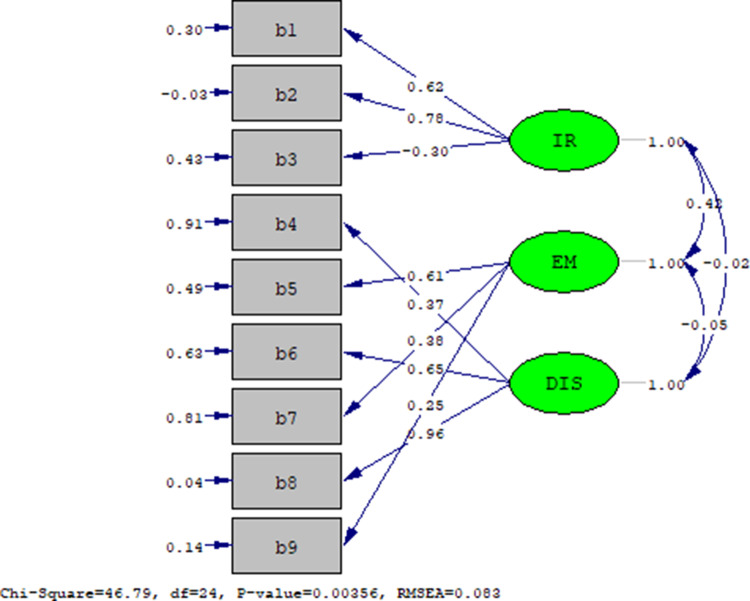
Confirmatory factor analysis of the 3-factor model of the HFT-BAS. IR = intimate relationship, EM = existential meaning, DIS = disorganization.

CFA was tested under the structural equation model. This model was evaluated using the unweighted least squares estimate in this study. The index for the overall goodness of fit of the measurement is as follows: chi-squared/degrees of freedom ratio (χ^2^/df ratio) (χ^2^/df) = (46.79)/(24) = 1.9, Goodness of Fit Index (GFI) = 0.93, Adjusted Goodness of Fit Index (AGFI) = 0.87, Non-Normed Fit Index (NNFI) = 0.87, Normed Fit Index (NFI) = 0.85, CFI = 0.92, Incremental Fit Index (IFI) = 0.92, Relative Fit Index (RFI) = 0.78, Parsimony Normed Fit Index (PNFI) = 0.57, Parsimony Goodness of Fit Index (PGFI) = 0.50, RMSEA = 0.08. The results validated the effectiveness of the 3-factor model with satisfactory goodness of fit to the HFT-BAS (Table 5; Fig. 1).

**Table 5.** Construct validity of the HFT-BAS (*n* = 139 (group B))

*Three-Factor model*	*X^2^/df*	*GFI*	*AGFI*	*NNFI*	*NFI*	*CFI*	*IFI*	*RFI*	*PNFI*	*PGFI*	*RMSEA*
HFT-BAS	46.79/24 =1.9	0.93	0.87	0.87	0.85	0.92	0.92	0.78	0.57	0.50	0.08

*X*^2^ = chi-square, df = degrees of freedom. GFI = Goodness of Fit Index, AGFI = Adjusted Goodness of Fit Index, *NNFI* = Non-Normed Fit Index, *NFI* = Normed Fit Index, *CFI* = Comparative Fit Index, *IFI* = Incremental Fit Index, *RFI* = *Relative Fit Index, PNFI* = Parsimony Normed Fit Index, *PGFI* = Parsimony Goodness of Fit Index. *RMSEA* = root mean square error of approximation.

## Discussion

Supporting FCs is a vital core function and topic of palliative care. Existing assessment tools are often cultural differences inherent in Western cultures. This study aims to develop a bereavement screening tool for FCs high risk in palliative care and evaluate its reliability and validity. This tool was derived from 4 main Western theories – FFGT, TTM, DPM, MRM, and considered the bereavement attitude of Eastern cultures to develop a brief and clinically tailored scale. EFA and CFA test results confirmed the scale has good reliability and validity. Constructing the HFT-BAS with 9 items and testing its reliability and validity align with our original purpose of designing this new scale.

After discussions with 12 experts in palliative care and a review of content validity, the study initially prepared a 9-question questionnaire. After 278 palliative inpatient FCs completed the questionnaire, 3 factors were extracted through EFA: “Intimate relationship,” “Existential meaning,” and “Disorganization.” A combination of an efficient 9-item scale, a cumulative explained variance of the 3 factors being 60.15%, and a large sample size provided good reliability of this scale.

In this study, the average scale score from the 278 participants was 21.1 ± 3.4, suggesting moderate bereavement (total score between 9 and 36, where higher scores indicate a high degree of bereavement due to higher intimate relationships). In the statistical test, 41% of FCS still accounted for 21 points and above as a high bereavement risk. A potential explanation could be that Taiwan consistently promotes shared palliative care decision-making among doctors, FCs, and patients in clinical practice. In Taiwan, primary caregivers are also important clinical decision-makers crucial to family members as they affirm the medical care provided by patients, caregivers, and medical staff (Hu and Wang 2021). Maintaining open communication among all stakeholders is reinforced by Symmons et al. where having a shared understanding of illness and death between FCs and patients can lower conflicts and increase harmony and comfort in palliative care (Symmons et al. 2022).

This study investigated the items-analysis, reliability, and inter-subscale correlations of the HFT-BAS. The item-total correlation coefficients ranged from 0.39 to 0.53 and demonstrated significant differences with total scores (except Q3). The correlation average levels higher than 0.30 among the items were adequate and thus, indicated that all items contributed to the scale (Polit and Beck 2012). After determining the number of (sub)scales, Cronbach’s alpha should be calculated for each (sub)scale separately. Cronbach’s alpha coefficient demonstrates the covariance level between the items of a scale (Terwee et al. 2007). In the present study, Cronbach’s alpha of the entire HFT-BAS scale was 0.70 while the 3 sub-scales were 0.50 to 0.82, values higher than 0.7 are ideal whereas values close to 0.60 are satisfactory (Curtis and Keeler 2021). The Cronbach’s alpha coefficient for the subscale “Intimate relationship” was less than 0.6 in our study, highlighting a lack of correlation among the items (Q3) on a scale. Conflicts exist high between FCs of terminal cancer patients in Taiwan (Hu and Wang 2021). Even though the lower Cronbach’s alpha of one of the subscales is 0.50, we still keep Q3 to the clinical quick evaluation in this subscale. We will more golden-standard bereavement scales to conduct the test and revise. Additionally, the coefficient is dependent upon the number of items in a scale (Polit and Beck 2012) and specific sample characteristics (Hays et al. 2005). Thus, the number of questions in the survey should be increased with more rigorous inclusion criteria.

Construct validity, which measures the goodness-of-fit of the HFT-BAS model, reported the following results: (*χ*2/*df*) = 1.9, GFI = 0.93, CFI = 0.92, and RMSEA = 0.08. The *χ*2 value is sensitive to the sample size and model characteristics, with larger sample sizes leading to higher *χ*2 values. A total value of <3 is recommended for (*χ*2/*df*) (Schmitt 2011). The GFI and CFI generally proposed values greater than 0.9. In addition, RMSEA, which verifies the rations between constructs and the items of the model, should be less than 0.08 (Hair et al. 2019). This study design demonstrated favorable results for adaptive construct validity in HFT-BAS.

In Taiwan, like other East Asian regions, medical clinical sites are often crowded, so medical personnel must provide medical care more efficiently. While some bereavement scales developed in the West have been adapted to East Asian culture and are considered reliable and valid, they are challenging to use for screening in clinical settings in Taiwan due to the number of questions and types of bereavement involved. For instance, the BRAT has 40 items, and the MMCGI has 50 items. Therefore, a new scale called HFT-BAS was developed with 9 questions to screen FCs experiencing high levels of grief quickly. This scale was designed to meet the needs of many medical clinical units in Taiwan. In addition, most medical care in Taiwan is provided in hospitals, so most FCs quit their jobs to focus on caring for patients. Therefore, the closeness and attachment between patients and FCs are relatively high, like some parts of the Caregiver Grief Scale (Meichsner et al. 2016), such as the fear of losing loved ones and the meaning to themself, etc., were similar. However, cultural differences are reflected in the interaction and support between family members in close, supportive, and conflicting relationships. In HFT-BA, instruments are better able to present.

## Conclusion

This study tested the HFT-BAS and is suitable for screening family members’ anticipated bereavement in palliative care in Taiwan. This 9-item scale has 3 subscales, “Intimate relationship,” “Existential meaning,” and “Disorganization,” and good psychometric properties. It can quickly evaluate the bereavement situation of FCs in palliative care and be incorporated into routine clinical practice to achieve earlier detection of bereavement status. Despite the HFT-BAS satisfying the reliability and validity criteria, 1 medical center may not accurately reflect FC situations in all Taiwanese urban and rural settings. Future studies should further employ golden-standard bereavement and grief scales to compare and use this scale in different regional hospitals, where its psychometric properties can be tested.

## Limitations

The present study has several limitations. First, it is a cross-sectional study, which, although suitable for reliability and validity analysis, does not consider how FCs’ status may change with time. Thus, future studies should implement a longitudinal study design. In addition, the scale cannot be extrapolated to all aspects of bereavement and grief, only focusing on clinical use and a gold standard for further conducted tests. Finally, the participants originated from a single medical center in Taiwan; thus, the results do not reflect all FCs experiencing bereavement in Taiwan.

## Data Availability

All research data are within the manuscript. Other data are available upon request from the corresponding author.
